# Supplementary material for: Local data for local programming: Results from an HIV biobehavioral survey among people who inject drugs in Livingstone, Lusaka, and Ndola, Zambia, 2021
Source: PLoS One. 2025 May 27;20(5):e0323919. doi: 10.1371/journal.pone.0323919 (PMC12111603; doi:10.1371/journal.pone.0323919)
Supplement: Attachment — Submitted filename: ETHICS~2.PDF [file pone.0323919.s001.PDF]

TROPICAL DISEASES RESEARCH CENTRE
Tel/Fax +260212 615444
P O Box 71769
tdrc.ethics@gmail.com
NDOLA, ZAMBIA

TDRC ETHICS REVIEW COMMITTEE
IRB REGISTRATION NUMBER : 00002911
FWA NUMBER : 00003729

TRC/C4/10/2019
17th October, 2019
Dr. John Mwale
National HIV/AIDS/STI/TB Council
P.O Box 38718
Lusaka

Dear Dr. Mwale

RE: ETHICAL APPROVAL OF STUDY PROTOCOL

Reference is made to the protocol entitled "Comments on the study entitled "**HIV and STI Biobehavioral Survey among High Risk Men in selected Towns in Zambia." STC/2019/10".**

On behalf of the Chairperson of the TDRC Ethics Review Committee (ERC), I wish to inform you that the Committee during its meeting held on 10th October 2019 in the TDRC Boardroom, reviewed your protocol and observed that protocol satisfied most of the key aspects of ethical approval.

However, it was noted that although you submitted a detailed Information Sheet and verbal consent form, these documents were not translated in the key local languages. Considering the category of your potential study participants and the sensitive nature of the study, the Committee felt that it was important that the study participants have a clear understanding of the study procedures and other concerns before enrolling in the study.

In view of the above, the Committee resolved to grant your study conditional ethics approval pending translation of these key documents.

Full approval will be granted as soon as the Committee reviews the translated versions of these key documents.

Yours faithfully,
TROPICAL DISEASES RESEARCH CENTRE

Edna Mwale. Simbayi
SECRETARY – TDRC Ethics Review Committee
cc: Chairperson – TDRC Ethics Review Committee

TROPICAL DISEASES RESEARCH CENTRE
[illegible]
2019
P O BOX 71769
NDOLA, ZAMBIA

TROPICAL DISEASES RESEARCH CENTRE
Tel/Fax +260212 615444
P O Box 71769
tdrc.ethics@gmail,com
NDOLA, ZAMBIA

TDRC ETHICS REVIEW COMMITTEE
IRB REGISTRATION NUMBER : 00002911
FWA NUMBER : 00003729

TRC/C4/10/2019
17th October, 2019
Dr. John Mwale
National HIV/AIDS/STI/TB Council
P.O Box 38718
Lusaka

Dear Dr. Mwale

RE: ETHICAL APPROVAL OF STUDY PROTOCOL

Reference is made to the protocol entitled "Comments on the study entitled "**HIV and STI Biobehavioral Survey among People who Inject Drugs and Female Sex Workers in selected Towns in Zambia**." **STC/2019/9"**.

On behalf of the Chairperson of the TDRC Ethics Review Committee (ERC), I wish to inform you that the Committee during its meeting held on 10th October 2019 in the TDRC Boardroom, reviewed your protocol and observed that protocol satisfied most of the key aspects of ethical approval.

However, it was noted that although you submitted a detailed Information Sheet and verbal consent form, these documents were not translated in the key local languages. Considering the category of your potential study participants and the sensitive nature of the study, the Committee felt that it was important that the study participants have a clear understanding of the study procedures and other concerns before enrolling in the study.

In view of the above, the Committee resolved to grant your study conditional ethics approval pending translation of these key documents.

Full approval will be granted as soon as the Committee reviews the translated versions of these key documents.

Yours faithfully,
TROPICAL DISEASES RESEARCH CENTRE

Edna Mwale. Simbayi
SECRETARY – TDRC Ethics Review Committee
cc: Chairperson – TDRC Ethics Review Committee

TRO[illegible] [illegible]AS[illegible] RESEARCH CENTRE
[illegible] 2019
P O BOX 71769
NDOLA, ZAMBIA

TROPICAL DISEASES RESEARCH CENTRE
Tel/Fax +260212 615444
P O Box 71769
tdrc.ethics@gmail.com
NDOLA, ZAMBIA

TDRC ETHICS REVIEW COMMITTEE
IRB REGISTRATION NUMBER : 00002911
FWA NUMBER : 00003729

TRC/C4/10/2019
31st October, 2019
Dr. John Mwale
National HIV/AIDS/STI/TB Council
P.O Box 38718
Lusaka

Dear Dr. Mwale

RE: ETHICAL APPROVAL OF STUDY PROTOCOL

Reference is made to the protocol entitled "Comments on the study entitled "**HIV and STI Biobehavioral Survey among High Risk Men in selected Towns in Zambia.**" **STC/2019/10".**

On behalf of the Chairperson of the TDRC Ethics Review Committee (ERC), I wish to inform you that the Committee reviewed all the translated versions of the Participants' Information Sheet and the Verbal Consent Forms and it is satisfied with the translations. Therefore, ethical approval has been granted based on the following conditions;

You are required to submit progress reports to the ERC twice a year. The Committee shall not provide renewal of ethical clearance for on-going projects in the absence of progress reports.

Should there be any protocol modifications or amendments or violations, you are required to notify the ERC and submit protocol amendments for approval.

You are now required to submit your protocol to the National Health Research Authority for final approval following the link: **https://www.nhra.org.zm** and submit a final report to the ERC Secretariat at the end of the study.

This approval is valid for the period 31st October, 2019 to 31st October, 2019

The Committee wishes you success in the execution of the study
Yours faithfully,

TROPICAL DISEASES RESEARCH CENTRE

Edna Mwale. Simbayi
SECRETARY – TDRC Ethics Review Committee
cc: Chairperson – TDRC Ethics Review Committee

TROPICAL DISEASES RESEARCH CENTRE
[illegible]
[illegible] 2019
P.O.BOX 71769
NDOLA, ZAMBIA

TROPICAL DISEASES RESEARCH CENTRE
Tel/Fax +260212 615444
P O Box 71769
tdrc.ethics@gmail,com
NDOLA, ZAMBIA

TDRC ETHICS REVIEW COMMITTEE
IRB REGISTRATION NUMBER : 00002911
FWA NUMBER : 00003729

TRC/C4/10/2019
31st October, 2019
Dr. John Mwale
National HIV/AIDS/STI/TB Council
P.O Box 38718
Lusaka

Dear Dr. Mwale

RE: ETHICAL APPROVAL OF STUDY PROTOCOL

Reference is made to the protocol entitled "Comments on the study entitled "**HIV and STI Biobehavioral Survey among Female Sex Workers and People Who Inject Drugs in selected Towns in Zambia**." **STC/2019/09".**

On behalf of the Chairperson of the TDRC Ethics Review Committee (ERC), I wish to inform you that the Committee reviewed all the translated versions of the Participants' Information Sheet and the Verbal Consent Forms and it is satisfied with the translations. Therefore, ethical approval has been granted based on the following conditions;

You are required to submit progress reports to the ERC twice a year. The Committee shall not provide renewal of ethical clearance for on-going projects in absence of progress reports.

Should there be any protocol modifications, amendments or violations you are required to notify the ERC and submit protocol amendments for approval.`

You are now required to submit your protocol to the National Health Research Authority for final approval following the link: **https://www.nhra.org.zm** and submit a final report to the ERC Secretariat at the end of the study.

This approval is valid for the period 31st October, 2019 to 31st October, 2019

The Committee wishes you success in the execution of the study
Yours faithfully,
TROPICAL DISEASES RESEARCH CENTRE

Edna Mwale. Simbayi
SECRETARY – TDRC Ethics Review Committee
cc: Chairperson – TDRC Ethics Review Committee

TROPICAL DISEASES RESEARCH CENTRE
[illegible]
[illegible] OCT 2019
P O BOX 71769
NDOLA, ZAMBIA
