# Supplementary material for: Local data for local programming: Results from an HIV biobehavioral survey among people who inject drugs in Livingstone, Lusaka, and Ndola, Zambia, 2021
Source: PLoS One. 2025 May 27;20(5):e0323919. doi: 10.1371/journal.pone.0323919 (PMC12111603; doi:10.1371/journal.pone.0323919)
Supplement: Attachment — Submitted filename: Response to Reviewers.docx [file pone.0323919.s002.docx]

PLoS ONE

03 March 2025

Response to Reviewers (Rebuttal Letter)

Dear Editor and Reviewers,

We sincerely thank you all for your thoughtful, meaningful, and informed feedback. We have done our absolute best to fully address all comments under the set of limitations we operated within for this work. Please find below the content of our rebuttal letter, where we have broken down all editor and reviewer input into separate comments that we numbered and addressed individually. Our responses are in red.

Again, we thank you for your time and effort and look forward to addressing any further concerns.

Sincerely,

Daniel J. Woytowich, DrPH, MPH

Corresponding author

[toj2@cdc.gov](mailto:toj2@cdc.gov)

When submitting your revision, we need you to address these additional requirements.

1. Please ensure that your manuscript meets PLOS ONE's style requirements, including those for file naming. The PLOS ONE style templates can be found at

https://journals.plos.org/plosone/s/file?id=wjVg/PLOSOne_formatting_sample_main_body.pdf and

https://journals.plos.org/plosone/s/file?id=ba62/PLOSOne_formatting_sample_title_authors_affiliations.pdf"

Author Response: Thank you. We have gone through and checked, and updated the formatting of the author list to denote groupings of those who contributed equally.

2. Please include a complete copy of PLOS’ questionnaire on inclusivity in global research in your revised manuscript. Our policy for research in this area aims to improve transparency in the reporting of research performed outside of researchers’ own country or community. The policy applies to researchers who have travelled to a different country to conduct research, research with Indigenous populations or their lands, and research on cultural artefacts. The questionnaire can also be requested at the journal’s discretion for any other submissions, even if these conditions are not met.  Please find more information on the policy and a link to download a blank copy of the questionnaire here: https://journals.plos.org/plosone/s/best-practices-in-research-reporting. Please upload a completed version of your questionnaire as Supporting Information when you resubmit your manuscript.

Author Response: This file has been included with our resubmission. The file name is “Inclusivity-in-global-research-questionnaire”.

3. We note that the grant information you provided in the ‘Funding Information’ and ‘Financial Disclosure’ sections do not match.

When you resubmit, please ensure that you provide the correct grant numbers for the awards you received for your study in the ‘Funding Information’ section.

Author Response: The Award Number is: 1NU2GGH002056. We have ensured the reported numbers match in the financial disclosure statement and funding information section.

4. Thank you for stating the following financial disclosure: 

 [This activity was supported by the US President’s Emergency Plan for AIDS Relief (PEPFAR) (https://www.state.gov/pepfar/) through the US Centers for Disease Control and Prevention (CDC) under the terms of cooperative agreement “Supporting sustainable surveillance systems among key populations and support the Government of Zambia to improve HIV-related services for KPs” (Prime Award No. 1NU2GGH002056). The findings and conclusions in this report are those of the author(s) and do not necessarily represent the official position of the funding agencies. ].

Please state what role the funders took in the study.  If the funders had no role, please state: ""The funders had no role in study design, data collection and analysis, decision to publish, or preparation of the manuscript.""

If this statement is not correct you must amend it as needed.

Please include this amended Role of Funder statement in your cover letter; we will change the online submission form on your behalf.

Author Response: Thank you. We have amended the above financial statement to also include the requested information, which has been included in its totality in the cover letter as requested. You can see the revised statement here as well:

“This activity was funded by the US President’s Emergency Plan for AIDS Relief (PEPFAR) (https://www.state.gov/pepfar/) through the US Centers for Disease Control and Prevention (CDC) under the terms of cooperative agreement “Supporting sustainable surveillance systems among key populations and support the Government of Zambia to improve HIV-related services for KPs” (Prime Award No. 1NU2GGH002056). The findings and conclusions in this report are those of the author(s) and do not necessarily represent the official position of the funding agencies. CDC employees, whose specific roles are outlined in the author contributions section, provided technical assistance and input into the study design, data collection, data analysis, decision to publish, and preparation of the manuscript.”

5. We note that you have indicated that there are restrictions to data sharing for this study. For studies involving human research participant data or other sensitive data, we encourage authors to share de-identified or anonymized data. However, when data cannot be publicly shared for ethical reasons, we allow authors to make their data sets available upon request. For information on unacceptable data access restrictions, please see http://journals.plos.org/plosone/s/data-availability#loc-unacceptable-data-access-restrictions. Before we proceed with your manuscript, please address the following prompts: **a)** If there are ethical or legal restrictions on sharing a de-identified data set, please explain them in detail (e.g., data contain potentially identifying or sensitive patient information, data are owned by a third-party organization, etc.) and who has imposed them (e.g., a Research Ethics Committee or Institutional Review Board, etc.). Please also provide contact information for a data access committee, ethics committee, or other institutional body to which data requests may be sent. **b)** If there are no restrictions, please upload the minimal anonymized data set necessary to replicate your study findings to a stable, public repository and provide us with the relevant URLs, DOIs, or accession numbers. Please see http://www.bmj.com/content/340/bmj.c181.long for guidelines on how to de-identify and prepare clinical data for publication. For a list of recommended repositories, please see https://journals.plos.org/plosone/s/recommended-repositories. You also have the option of uploading the data as Supporting Information files, but we would recommend depositing data directly to a data repository if possible. Please update your Data Availability statement in the submission form accordingly.

**Author response:** Thank you for this comment. The underlying data for this study are owned by the Zambian Ministry of Health and the National AIDS Council. Due to the sensitive nature of the data, which involve key populations that face legal and social risks, there are strict ethical and legal restrictions on data sharing. These restrictions are mandated by the institutional review board (IRB) requirements to protect participant privacy and confidentiality. For these reasons, the de-identified data can only be accessed upon request and after approval by the relevant Zambian health authorities. Researchers interested in accessing the data should contact:

Dr Kebby Chongwe Musokotwane

Director General

National HIV/AIDS/STI/TB Council

Plot 315, Independence Avenue

PO Box 38718

Lusaka, Zambia

Tel: +260-211-255044

[KMusokotwane@nacsec.org.zm](mailto:KMusokotwane@nacsec.org.zm)

Requests will be evaluated to ensure compliance with ethical standards and participant safety.

6. Please review your reference list to ensure that it is complete and correct. If you have cited papers that have been retracted, please include the rationale for doing so in the manuscript text, or remove these references and replace them with relevant current references. Any changes to the reference list should be mentioned in the rebuttal letter that accompanies your revised manuscript. If you need to cite a retracted article, indicate the article’s retracted status in the References list and also include a citation and full reference for the retraction notice.

Author Response: Thank you. We have ensured our reference list is complete and correct.

**Additional Editor Comments:**

**1. Introduction (lines 82 - 84). The objective does not mention assessment or presentation of results around syphilis, HBV or HCV (STI and BBV), so consider noting these too.**

Author Response: Thank you for noticing this. We have added these components into the relevant lines of the Introduction Section of the revised manuscript.

**2. Introduction. The provision and availability of needle and syringe programmes (NSP) and opioid agonist maintenance therapy (OAMT) and treatment of stimulant dependence in Zambia should be briefly described.**

Author Response: Thank you. The following basic information has been added into the 3^rd^ paragraph of the introduction section:

*“In addition, there are no PEPFAR or government-sponsored Opioid Agonist Maintenance Therapy (OAMT) or Needle and Syringe Programs (NSPs) available for the treatment of stimulant dependence in Zambia.”*

We have also added information to the discussion section about some work that is happening to create policy, guidance, and institutional space for the hopeful enabling of methadone treatment in Zambia. This addition to the discussion section has been explained in more detail in our response to the editor’s later comment about NSP and OAMT in #11 below.

**3. Methods (lines 92). Insert a short description about each study site for readers who may not be familiar with Zambia.**

Author Response: The following descriptions have been added to the methods section:

*“Livingstone is in Southwest Zambia on the border with Zimbabwe and has a high influx of visitors for Victoria Falls, but nonetheless has the smallest population of the three survey sites. Lusaka is in Central Zambia, is the nation’s capital, and has the largest population of the three sites. Ndola lies roughly 300 km to the north of Lusaka near the border with the Democratic Republic of the Congo.”*

**4. Findings. The findings section (notably descriptions) is a bit long in parts. Aim to focus on key findings, and reduce some descriptions of socio demographic characteristics, and refer to the table.**

Author Response: Thank you, we agree. The language throughout the whole ‘Results’ section has been streamlined and shortened while some of the repetitive/less important descriptions have been reduced or omitted.

**5. Findings. Table 1. Insert the % for all rows, where relevant (currently only done for some).**

Author Response: We agree this helps with the standardization of the table. This has been done.

**6. Findings. Unless I missed it, I did not see the description of the type of drugs injected. This is critical to assess risk, prevention and interventions.**

Author Response: The most common type of drugs injected across the three sites has been added to Table 3 and the corresponding paragraph in the findings. There will be another manuscript published from another partner research group that goes into more detail about injection drug practices, prevention, and interventions in this PWID population, which is why we originally didn’t include it here. However, we agree that this brief background will help contextualize the 95-95-95 progress we are reporting on. Thank you for this suggestion.

**7. Table 3. Median duration of injecting. Check the median and p25 and p75 - I would expect the median to lie between these, but does not seem to be the case (e.g., Ndola median is 3.0 years, but IQR is 6 - 10 year).**

Author Response: Thank you so much for noticing this, not sure how it slipped by after so many reviews on our end. These medians and their p25 and p75’s have been recalculated and have now been fixed in Table 3. These numbers have been corrected within paragraphs where median duration of injecting was reported or referenced as well.

**8. Insert n for anti-HCV results and HCV PCR for sample in table and findings description.**

Author Response: Thank you for your comment. With such small sample sizes, it was the team’s policy to put an asterisk in place of n’s that were 5 or less in order to protect this vulnerable population from being identified through data triangulation. In any tables in which an asterisk is shown, the asterisk is described as such in the table’s footnote. All of the local authors and advisors from the vulnerable groups themselves agreed with this decision. It was a deliberate choice so they could not be identified by an employer, family member, etc. since stigma and bias is so common with this group.

**9. Findings around awareness of OAMT - this would only apply to people with opioid dependence, but it is not clear what drugs people injected. Also, Table 3 notes " aware of any drug treatment programs" - this is too broad to reflect insights into OAMT. So if questions on OAMT were asked, please include. If not, this could be noted in the limitations.**

Author Response: Thank you, this is great insight. In response to this and your previous comment about not knowing which types of drugs people injected, we included in Table 3 the most common types of drugs injected across the three sites, so it is our hope this provides some helpful context. In addition, an asterisk is included in the ‘Awareness of any drug treatment programs’ variable of Table 3 corresponding to a footnote that provides further details by stating that the variable includes outpatient, inpatient, residential, detox, and methadone maintenance drug treatment programs. Unfortunately, the original survey question written years ago didn’t tease out awareness based on type of program into any more detail than this, so this has been noted in the Limitations section too as you suggested:

*“Our variable on awareness of drug treatment programs (including outpatient, inpatient, residential, detox, and methadone maintenance therapy) is of limited value since OAMT and NSPs are currently not available in Zambia, however the low awareness observed indicates work must be done to increase awareness on the benefits of these programs if they are to be maximally successful if and when they are approved in Zambia.”*

**10. Findings. Why no findings of needle reuse/ sharing? This is the most important risk factor for PWID? (Unless I missed it). This is key to include, as it is a UNAIDS GAM indicator. Please include these variables.**

Author Response: An indicator regarding frequency of needle reuse in the last 6 months has been included in Table 3 and the corresponding paragraph. We definitely agree with your statement but originally didn’t include this variable in the manuscript because survey designers wrote this question subjectively. The original survey question had response categories of ‘Never’, ‘Rarely’, ‘Half of the time’, ‘Most of the time’, ‘Always’, and ‘Don’t know/No answer’. For the purposes of the paper, these categories have been recoded into ‘Never’, ‘At least once’, and ‘No answer’ to make it as objective and accurate as possible given the constraints in how this question was originally constructed.

**11. Discussion. Among PWID, the primary HIV prevention interventions are NSP and OAMT, these should be foregrounded. These interventions are more important for PWID than PrEP, yet there is much emphasis on PrEP. Similarly, the implications for policy and practice, NSP and OAMT need to be much higher priorities than PrEP, but the framing does not align with emphasis on the issues that are particular to PWID. There is mention of MAT clinics, but what about NSP? This is critical, as many African countries are resistant to NSP, but without NSP HIV prevention interventions for PWID will fail to be effective.**

Author Response: We agree NSPs and OAMT are very important for PWID. Unfortunately, as discussed above in our response to your other comment regarding the introduction section, they are currently not available in Zambia, whereas PrEP is. However, there is work going on regarding getting OAMT approved so we have added some detail about this into our “Implications for Program and Policy” subsection of the Discussion. Please see the information that has been added to the discussion section below:

*“…Preventive resources like PrEP can be efficiently offered in these facilities as well (59), along with OAMT (60)) and NSPs. Results from the 2021 BBS have already propelled the establishment of a PWID task force with representation from government and non-governmental agencies that is working to establish MAT clinics while the police and Home Affairs Ministry offer basic rehabilitative services to the public in Lusaka. The PWID task force is also in the process of creating national policy regarding methadone approval in Zambia. In preparation for its approval, University Teaching Hospital in Lusaka is preparing space for a MAT clinic, but at the current time is only offering services like HIV testing, treatment, and wound care to attract PWID clientele for when OAMT becomes approved. Again, OAMT and NSPs, if approved, would likely serve as a strong facilitator to getting PWID into the HIV testing and treatment pathway. To this end, OAMT may be most impactful in Livingstone and Lusaka where Heroin is by far the most commonly reported drug injected, as opposed to Ndola where Trihexyphenidyl is most common; whereas NSPs may be most impactful in Livingstone and Ndola, where PWID reported much higher rates of injecting with a used needle than Lusaka. ….”*

We have also added wording to our limitations section about the application of our “Awareness of drug treatment programs” variable:

*“Our variable on awareness of drug treatment programs (including outpatient, inpatient, residential, detox, and methadone maintenance therapy) is of limited value since OAMT and NSPs are currently not available in Zambia, however the low awareness observed indicates work must be done to increase awareness on the benefits of these programs if they are to be maximally successful if and when they are approved in Zambia.”*

We have also added in a statement about the benefits of OAMT and NSPs into our conclusion which is described in more detail in our response to #13 below.

**12. Discussion. The low HBV and (HCV?) prevalence is encouraging, and the implication for policy could also be to continue active surveillance and testing in these high risk groups to control the epidemic, and have access to HCV treatment early, before there is an epidemic, like is happening in other countries (e.g., South Africa).**

Author Response: Thank you for the suggestion. While we kept it to a minimum since we wanted to maintain focus on HIV and the UNAIDS cascade and since another research group associated with this project will likely be completing a manuscript where HBV and HCV are more of a central focus, we did add in the brief statement and reference below (regarding HCV point-of-service care in South Africa) to the end of the discussion section:

*“Also encouraging was the low HBV and HCV prevalence across all three sites. Active HBV/HCV surveillance and testing should continue within the high-risk PWID community, while consideration can be given to streamlined treatment models, such as point-of-service care (61).”*

**13. Conclusion. Reflection on the important gap of NSPs seems to be missing. NSPs should be framed as being the link to access HTS and then enter the treatment pathway.**

Author Response: We agree this is a very important point. The majority of the information we added concerning the hopeful policy creation and approval of OAMT and NSPs in Zambia is contained in the revisions/additions we added into the discussion section in response to your above comments about OAMT/NSPs. However, we did add some detail about OAMT and NSPs being a link to testing into our conclusion statements as well. Please find the amended text below:

*“Improved counseling by HCPs; the establishment of community-based PWID-friendly HIV, OAMT, and NSP clinics that can serve as another link for helping PWID access HIV testing; and better coordination between HCPs, peer educators, and outreach workers in proactively contacting, counseling, and guiding PWID through the HIV prevention and care continuum may help improve these outcomes.”*

**Reviewer #1:**

**Abstract**

**14. Lines 38-39 – don’t need to mention both HIV and HIV viral load testing.**

Author Response: Thank you for mentioning this, this has been amended in the revised manuscript.

**15. Results – find the (1st 95) confusing, same for 2nd and 3rd – link to targets unclear. For those less familiar with HIV targets, it may be helpful to say what the targets are when they’re being discussed (and elsewhere in the paper).**

Author Response: Thank you for the suggestion, we have gone back and forth a lot with how to construct the abstract. Given the 300-word limit, we were unfortunately limited with how much detail we could go into for background explanations, which really increase the word count rapidly. However, in response to your suggestion we did add some more detail about the UNAIDS 95’s into the parenthesis of the Abstract’s Results section. Also, we have explained the 95’s and their interpretation in more detail in the second paragraph of the introduction section. We thank you for your comment and hope these changes have clarified these details.

**Manuscript**

**16. Line 96 – the ‘prevalence of VLS’ may be better described as the proportion of people living with HIV with VLS**

Author Response: Thank you for this suggestion. Your suggested language has been incorporated into the revised manuscript.

**17. Lines 102-103 – not sure the data on sample size calculations are needed if they were missed by such a large margin. If the authors want to include the calculation, more details are needed on the factors limiting study size (costs/ time constraints etc).**

Author Response: Thank you for this comment. Our agency required that we include this information and therefore adhere to the STROBE-RDS study reporting checklist: [Microsoft Word - STROBE-RDS Checklist_ToEquator.doc](https://www.equator-network.org/wp-content/uploads/2013/09/5204_STROBE-RDS-Checklist.pdf). We did however provide a bit more information on the limiting factors and so this section has been reworded as follows:

*”Therefore, a target sample size of 780 PWID for each city was calculated to obtain a sample size of 195 HIV-positive PWID participants per city assuming an HIV prevalence of 25%. However, this was determined to be unattainable after consultation with local stakeholders supporting PWID in Zambia due to workforce and funding limitations. Therefore, this target was divided across the sites based upon the size of the PWID population in each city (Livingstone, n=215; Lusaka, n=350; Ndola, n=215).”*

**18. Lines – pre-test counselling for HIV/syphilis is mentioned, was this also done for HBV/HCV, or are there no national guidelines on this?**

Author Response: Thank you for catching this. HBV and HCV should have been included in that sentence about pre-test counseling as HBV/HCV was also part of the pre-test counseling as dictated by national guidelines. The sentence has been amended as follows:

*“Participants who consented to being tested received pre-test counseling for HIV, active syphilis, HBV, and HCV that followed national guidelines (33).”*

**19. Line 142 – Might be useful to state where the Tropical Diseases Research Centre (TDRC) laboratory is based**

Author Response: TDRC is in Ndola. This detail has been added to the revised manuscript. Thank you for bringing up this point of clarification.

**20. Results lines 196-320, Tables 2-4 – this section contains a large amount of data in well-presented tables but overall, is very descriptive with the paragraphs describing the data from each table for each location. I would like to see more comparison between the sites in this section – ie were people younger/older at one site? Were there different risk factors at each site? Was one site better at getting PWID onto ART? Some of this is mentioned in the discussion but is perhaps better placed in the results section. Some statistics to highlight differences between the sites would be informative and help to summarise the data.**

Author Response: Thank you for this comment. The language throughout the whole ‘Results’ section has been streamlined while some of the repetitive/less important descriptions have been reduced along with bolstering the information with some comparisons. We were hesitant to heavily report statistical differences across sites because of the generally wide confidence intervals we obtained due to our limitation of relatively small sample sizes. We also wanted to maintain one of our original goals of this paper which was to focus on the site- and context-specific nature of the data, and so left major comparisons out until the Discussion section where we feel they fit in better with our ‘Implications for Program and Policy’ section which is wholly devoted to a synthesis of results across the sites and comparisons between them. In addition, in our newly revised manuscript, we added a considerable amount of information to the ‘Implications for Program and Policy’, ‘Limitations’, and ‘Conclusion’ sections in response to other reviewers, much of which added context and detail to our synthesis of information from across the sites. Lastly, we have addressed in the ‘Limitations’ section why we didn’t include results from statistical modeling, which was due again to the small sample sizes, large confidence intervals, and contextual and societal differences across sites that we feel would greatly risk making direct statistical comparisons and conclusions drawn from them spurious. In addition to the information, basic comparisons, and synthesis across sites we added to the ‘Results’, ‘Discussion’, and ‘Conclusion’ section, we added information to the ‘Introduction’ section as well and variables to the Tables (e.g., Most common drugs injected, frequency of injecting with a used needle, etc.) that all add to the totality of what we’re presenting.

**21. Discussion – it would be useful to get some idea of how widespread injection drug use is in Zambia, and particularly in Ndola where the HIV prevalence among PWID is very high.**

Author Response: Thank you for this query. Unfortunately there was no recent published information on injection drug use prevalence throughout Zambia; but, while we didn’t want to go into too much detail about population size estimations since another research group will be publishing on the methods and results for calculating these, we did provide the following details in the portion of the ‘Methods’ section that presents background information on the survey sites:

*“In the six months prior to the survey, the consensus estimate among 15-64-year-olds for the PWID population size in Livingstone, Lusaka, and Ndola was 0.9% (95% CI: 0.7, 1.5), 0.2% (95% CI: 0.1, 0.5), and 0.6% (95% CI: 0.4, 0.7), respectively.”*

**Reviewer #2:** 


**22. General comment:** **Are there any prevention services available for PWID specifically in Zambia, either NSP or OAMT? The instrument asked about OAMT awareness but were there questions about uptake? Or where needles were obtained?**

Author Response: Thank you. Unfortunately, there are no PEPFAR or Zambian government-sponsored OAMT and/or NSP programs in Zambia, which we have now explained in the ‘Introduction’ section. More substantially, we have added information to the “Implications for Program and Policy” subsection of the Discussion about work that is currently happening to create policy for approval, guidance, and institutional space for the hopeful enabling of OAMT treatment in Zambia. Please see the information that has been added to the discussion section below:

*“…Preventive resources like PrEP can be efficiently offered in these facilities as well (59), along with OAMT (60)) and NSPs. Results from the 2021 BBS have already propelled the establishment of a PWID task force with representation from government and non-governmental agencies that is working to establish MAT clinics while the police and Home Affairs Ministry offer basic rehabilitative services to the public in Lusaka. The PWID task force is also in the process of creating national policy regarding methadone approval in Zambia. In preparation for its approval, University Teaching Hospital in Lusaka is preparing space for a MAT clinic, but at the current time is only offering services like HIV testing, treatment, and wound care to attract PWID clientele for when OAMT becomes approved. Again, OAMT and NSPs, if approved, would likely serve as a strong facilitator to getting PWID into the HIV testing and treatment pathway. To this end, OAMT may be most impactful in Livingstone and Lusaka where Heroin is by far the most commonly reported drug injected, as opposed to Ndola where Trihexyphenidyl is most common; whereas NSPs may be most impactful in Livingstone and Ndola, where PWID reported much higher rates of injecting with a used needle than Lusaka. ….”*

We have also added wording to our limitations section about the application of our “Awareness of drug treatment programs” variable:

*“Our variable on awareness of drug treatment programs (including outpatient, inpatient, residential, detox, and methadone maintenance therapy) is of limited value since OAMT and NSPs are currently not available in Zambia, however the low awareness observed indicates work must be done to increase awareness on the benefits of these programs if they are to be maximally successful if and when they are approved in Zambia.”*

We have also added in a statement about the benefits of OAMT and NSPs into our conclusion.

**23. In comparing the results in the article with the IBBS report found online the values do not match (e.g. HIV prevalence in Lusaka is 21.9% in the article vs 21.3% in the report; the 95’s for Ndola in the article are 60.2%-100%-90.2% while in the report there are 61.9%-100%-83.7%... ). Could these discrepancies be clarified for readers who may look at the report, too?**

Author Response: Yes, you’re correct, the estimates in the IBBS report are slightly different than ours. The reason for this is because all our analyses’ estimates were computed using weights generated with Giles’ Sequential Sampling (Giles’ SS) estimator along with bootstrapping in RDS-A software, which is slightly more accurate for datasets like ours. In the IBBS report, they used SAS survey procedures to estimate proportions and confidence intervals with Taylor Series Variances. Point estimates computed in this way are usually very close to the bootstrap estimates, but the confidence intervals can vary by several percentage points, especially in small cells. This resulted in the slightly more robust and accurate estimates in this manuscript as opposed to the IBBS report, which was done several years ago.

**24. Line 34: “progress of PWID”…is it progress of the PWID themselves or the epidemic response for and by them? It reads awkwardly as written.**

Author Response: This is a good point, thank you for picking it up. We have amended that sentence in the abstract to read as follows:

*“We report here on the progress within the PWID communities of Livingstone, Lusaka, and Ndola, Zambia towards the Joint United Nations Programme on HIV/AIDS (UNAIDS) 95-95-95 targets.”*

We hope this is an improvement but are happy to amend further.

**25. Line 59: “Whereas the world’s general population has an HIV prevalence of 0.7%...”
It would be worth to specify if this refers to all ages, 15+ or any other age range. The same applies to all the values related to the general population in the article (e.g. Line 61).
Also, the value would need bounds, kindly add them, as well as all the rest of the values in the article.**

Author Response: Thank you for catching this. The age ranges and 95% CI bounds have been added for these values.

**26. Line 63: “…in which 19.6% of SSA PWID participate.”
The source from where the data come from specify that the people have participated recently.**

Author Response: Thank you, we’ve added the 95% CI to this statistic in accordance with your previous comment and changed the language to specify that the source referred to the previous year as ‘recent’. The sentence now reads as follows:

*“HIV transmission occurs between PWID when injecting equipment is shared and from PWID to others through activities like sex work (16), in which 19.6% (95% CI: 11.1, 31.3) of SSA PWID have recently (within a year) participated (11).”*

**27. Line 68: “… defined as HIV RNA of <1000 copies/mL during the most recent VL test”.
The source for this is: 2016 World Health Organization (WHO) Consolidated guidelines on the use of antiretroviral drugs for treating and preventing HIV infection. Kindly update it.**

Author Response: Thank you for bringing this to our attention. The phrase about HIV RNA mentioned in your comment has been attributed to the source you mentioned.

**28. Lines 96-97: “…people living with HIV in Zambia was 59.2% (19).”
The correct citation for this seems to be "Ministry of Health, Zambia. Zambia Population-based HIV Impact Assessment (ZAMPHIA) 2016: Final Report. Lusaka, Ministry of Health. February 2019".**

Author Response: Thank you for noticing this and bringing this to our attention. This citation has been corrected.

**29. Line 101: “…assuming an HIV prevalence of 25%”.
Why assume a prevalence of 25% if the evidence shown on the introduction showed 15.2% globally and 11.2% in SSA?**

Author Response: Thank you. The protocol was written and informed by local stakeholder consultations almost 5 years ago now at this point, which were focused on best estimations being that this was the first PWID HIV study in these locations. The numbers cited in the introduction (15.2 and 11.2) are from a much newer source of information published in 2023.

**30. Line 236: “…healthcare because they inject drugs…”
Please specify that they have being treated unfairly or denied healthcare by healthcare practitioners.**

Author Response: This has been added and fixed. Thank you for noticing this.

**31. Line 253: “…injected for a median duration of 3 years (IQR: 6-10).”
Why the IQR do not have decimal numbers? Please consolidate with the rest of the article.**

Author Response: This has been fixed throughout the paper, thank you for noticing.

**32. Line 266: “…with HIV, 70.8% (95% CI: 56.8, 85.1) were virally suppressed”. & Line 294 “…and 58.0% (95% CI: 27.8, 88.3) of…”
Why to use the cascade here if in the abstract and introduction the reference is the 95-95-95?**

Author Response: You are definitely right that the focus of the paper is the conditional UNAIDS 95’s, so we discuss ART and VLS within that context in the Abstract, Introduction, in the “Population-level 95-95-95 Progress” subsection of the Results, Figure 1, and in the Discussion. However, we reported VLS in the lines you referenced and Table 4 as a proportion of all those with HIV (unconditional) in order to provide the reader with a quick and simple snapshot of the risk profile in the three study sites (e.g., simply, how many of the PLHIV across the three sites were suppressed, and therefore less risk to others).

**33. Lines 273-274: “…from peer educators and/or outreach workers (47.2%), internet (11.9%), television (8.5%), radio (2.9%), and/or friends (31.9%).” & Lines 288-289 “…workers (47.1%), internet (9.1%), television (35.9%), radio (56.6%), and/or friends (37.3%).”& Lines 303-304 “…workers (31.0%), the internet (5.7%), television (5.0%), radio (6.5%), and/or friends (13.8%).”
Why not adding IC 95% on these values?**

Author Response: We have amended this issue and placed the 95% Confidence Intervals in the paragraph. Thank you.

**34. Line 277: “…Number of Hepatitis B cases…”.
Could you kindly add that Hepatitis C cases too?**

Author Response: Yes, this is a good suggestion. The Hepatitis C cases for Table 4 have been added at the end of the Livingstone, Lusaka, and Ndola paragraphs.

**35. Line 284: “… because they did not feel at risk whereas 22.8% (7.2, 38.2) did…”
Please add that numbers within brackets correspond to 95% CI.**

Author Response: Thank you for noticing, this has been fixed.

**36. Line 332: “…forced sex, and lower awareness of non-injectable alternatives present unique challenges.”
Please specify where this sentence comes from. It hasn't been introduced through the article.**

Author Response: The high rate of forced sex in Livingstone is shown in Table 2 and introduced in the Livingstone subsection of the “Sociodemographic and sexual history characteristics” part of the ‘Results’. Regarding the “non-injectable alternatives”, you are right. We had taken that variable out and instead presented a similar variable entitled “Aware of any drug treatment programs” which is introduced in Table 3 and in the Livingstone subsection of the “Injection drug use and history of stigma, discrimination, and criminalization” part of the ‘Results’. Now, we have amended this phrasing in the portion of the ‘Discussion’ section that you have pulled out above so it now reads: *“…higher prevalence of forced sex, and lower awareness of drug treatment programs present unique challenges*.” We appreciate you catching this. The wording in the ‘Discussion’ section is now consistent with how these topics were introduced in the ‘Results’ section.

**37. Line 343: “…(13.2%), where Livingstone is located (19).” & Line 344 “…women (5.9%) and men (1.8%) of the country-wide…” & Lines 354-355-356 “…slightly higher than women (5.9%) and men (1.8%) of Zambia’s general population of the same age group but lower than the all-age prevalence in the broader Lusaka province’s general population (14.4%) (19)” & Lines 373-374: “…Copperbelt province (11.9%) (19), where Ndola is located. It was also higher than women (12.0%) and men (3.1%) in…”
Specially interesting to include IC 95% here to see if those overlap with the results found.**

Author Response: Unfortunately, in this resource (The 2016 Zambia Population-based HIV/AIDS Impact Assessment (ZAMPHIA)), confidence intervals were not reported by province, age, and sex. We spent time trying to find these numbers in source documents for the ZAMPHIA and in other sources altogether but were not able to find them. We apologize for this inconvenience but we included all the confidence intervals we could.

**38. Line 362: “…discovering they inject drugs, and almost 8.0%...”
Is 8 percentual points, may be confusing, perhaps helpful to clarify.**

Author Response: Thank you for bringing this point of clarification to our attention. The phrase has been amended as follows: *“…and almost 8.0 percentage points less PWID had been reached by peer educators and/or outreach workers.”* We hope this is more clear.

**39. Line 368: “… avoidance of healthcare,…”
Please include that it is “high frequency of” avoidance of healthcare.**

Author Response: Thank you. This phrase has been amended as follows: *“high frequency of healthcare avoidance,…”*

**40. Line 376: “… almost double the prevalence…”.
Is most of the double of each of the other two cities.**

Author Response: You’re right, it is more than double, not almost double. This has been fixed. Thank you for noticing.

**41. Lines 456-457: “…was relatively low, awareness of the benefits of opioid agonist therapy was low…”
This was not mentioned before in the article, can be specified where this come from?**

Author Response: This is a similar issue to what you mentioned above in your comment in #36 about Line 332 and again, we thank you for noticing this. In an effort to streamline the paper, the variable about risk perception was taken out previously and we had neglected to omit this phrase from this sentence that you pulled out from the ‘Conclusion’. Also, we switched the “awareness of the benefits of opioid agonist therapy” variable for a variable entitled “Aware of drug treatment programs”. This has been fixed as well in the sentence you pulled out from the ‘Conclusion’. The sentence is now consistent with what was presented previously in the paper and reads as follows: *“Only about 60% of PWID had been in contact with peer educators and/or outreach workers, awareness of drug treatment programs was low, needle sharing was common, many PWID who had been tested for HIV did so more than a year before the survey, and an appreciable number of respondents avoided healthcare due to the fear of someone discovering their injection drug use.”*

6. PLOS authors have the option to publish the peer review history of their article ([what does this mean?](https://journals.plos.org/plosone/s/editorial-and-peer-review-process#loc-peer-review-history)). If published, this will include your full peer review and any attached files.


If you choose “no”, your identity will remain anonymous but your review may still be made public.


**Do you want your identity to be public for this peer review?** For information about this choice, including consent withdrawal, please see our [Privacy Policy](https://www.plos.org/privacy-policy).

Reviewer #1: No

Reviewer #2: **Yes:** Keith Sabin

[NOTE: If reviewer comments were submitted as an attachment file, they will be attached to this email and accessible via the submission site. Please log into your account, locate the manuscript record, and check for the action link "View Attachments". If this link does not appear, there are no attachment files.]

While revising your submission, please upload your figure files to the Preflight Analysis and Conversion Engine (PACE) digital diagnostic tool, <https://pacev2.apexcovantage.com/>. PACE helps ensure that figures meet PLOS requirements. To use PACE, you must first register as a user. Registration is free. Then, login and navigate to the UPLOAD tab, where you will find detailed instructions on how to use the tool. If you encounter any issues or have any questions when using PACE, please email PLOS at <a href="mailto:figures@plos.org">figures@plos.org. Please note that Supporting Information files do not need this step.

*In compliance with data protection regulations, you may request that we remove your personal registration details at any time.* [*(Remove my information/details)*](https://www.editorialmanager.com/pone/login.asp?a=r)*. Please contact the publication office if you have any questions.*
